# Using MRI Measurement to Improve Accuracy of Femoral Component Sizing in Oxford Unicompartmental Knee Arthroplasty

**DOI:** 10.3390/jcm10184284

**Published:** 2021-09-21

**Authors:** Cheng-Pang Yang, Ying-Chieh Lai, Chen-Te Wu, Kung-Tseng Hung, Yi-Sheng Chan, Alvin Chao-Yu Chen, Kuo-Yao Hsu

**Affiliations:** 1 Department of Orthopedic Surgery, Division of Sports Medicine, Chang Gung Memorial Hospital and Chang Gung University College of Medicine, Linkou, Taoyuan 333, Taiwan; ronnie80097@gmail.com (C.-P.Y.); st900567@gmail.com (K.-T.H.); yschan512@gmail.com (Y.-S.C.); alvin_ortho@yahoo.com (A.C.-Y.C.); 2 Bone and Joint Research Center, Chang Gung Memorial Hospital, Linkou 333, Taiwan; 3 Comprehensive Sports Medicine Center, Linkou Chang Gung Memorial Hospital, Linkou 333, Taiwan; 4 Department of Medical Imaging and Intervention, Chang Gung Memorial Hospital, Chang Gung University College of Medicine, Taoyuan 333, Taiwan; cappolya@gmail.com (Y.-C.L.); melik@cgmh.org.tw (C.-T.W.)

**Keywords:** unicompartmental knee arthroplasty, femoral component size, MRI measurement, anteromedial osteoarthritis

## Abstract

Unicompartmental knee arthroplasty (UKA) can achieve better kinematics and faster recovery than total knee arthroplasty. The Phase III Oxford UKA system has five sizes of femoral components to approximate the normal knee geometry. However, these different sizes may also induce problems, such as the misselection of component size. Different criteria have been proposed to predict the ideal size preoperatively. However, no single method can be applied universally. Therefore, this study aimed to develop a preoperative measurement using knee magnetic resonance imaging (MRI) to predict femoral component size. A total of 68 patients who underwent UKA were investigated from June 2019 to April 2020. 16 knees using a different MRI protocol were excluded. We developed an MRI measurement method to determine femoral size instead of gender- and height-based methods. The accuracy of different methods was compared using postoperative true lateral view radiographs. Three different kinds of gender- and height-based criteria, preoperative templating and intraoperative spoon measurement were compared. The accuracy of MRI measurement was 90.3%. Therefore, a significant difference was found between MRI measurements and all other methods, such as templating or gender- and height-based methods. In conclusion, the MRI measurement method can be concluded to accurately predict femoral component size in UKA. This method could be used regardless of different ethnic groups, individual knee geometry, or soft tissue tension.

## 1. Introduction

Oxford unicompartmental knee arthroplasty (OUKA) is a successful treatment option for anteromedial osteoarthritis. In 1998, Phase 3 instrumentation and implants were introduced and have become increasingly well known. Compared to total joint arthroplasty (TKA), OUKA approximates knee kinematics more closely and is associated with a less invasive approach, quicker recovery and higher patient satisfaction [1,2,3,4,5]. Phase 3 has five femoral components size: extra small (XS), small, medium, large and extra-large (XL). The size of Phase 1 and 2 components is equal to that of the medium in Phase 3. These different sizes enable similarity of the original anatomy. However, the Phase 3 implants has led to some potential problems, like using an incorrect size [6,7,8,9,10].

Several methods have been used to predict the size of the femoral component, such as preoperative template, intraoperative sizing spoon, estimation from the patient’s height and gender [6,7,11,12,13]. The original height- and gender-based guidelines have been used out of experience in Caucasians [6]. Later, modified versions were published by Tu et al. in the Chinese population and Malhotra et al. in Indian patients [11,12]. However, due to the varied anatomy in different populations, no single guideline can be applied to all populations.

On the contrary, the role of magnetic resonance imaging (MRI) in Oxford unicompartmental knee replacement is still debated [14,15]. MRI has once been proposed to assess the suitability of UKA. However, Hurst et al. suggested that patients who underwent UKA despite MRI contraindications had no difference in clinical outcomes, as confirmed by MRI [16]. Furthermore, MRI examinations cost more time and money. Recently, a study using radiographic aid decision protocol for UKA and suggested the use of MRI in a situation where no medial bone-on-bone lesion was noted [17].

Our goal is to establish a patient-specific method based on MRI to correctly predict femoral component size and compare the results with different guidelines and spoon measurement. To our knowledge, this is the first report of an MRI-based measurement to determine femoral component size.

## 2. Materials and Methods

We consecutively collected a series of 68 knees with isolated osteoarthritis (OA) of the medial compartment implanted with the Oxford phase 3 UKA (Oxford Partial Knee, Biomet Orthopedics, Bridgend, UK) between June 2019 and April 2020. The UKA indication established by Goodfellow et al. was used [18], which required full-thickness cartilage loss in the medial compartment on both the femur and tibia. The patients’ height and gender were recorded. All patients underwent preoperative MRI examination. MRI was arranged to evaluate cartilage loss, anterior cruciate ligament (ACL) degeneration and meniscal root lesion. A total of 16 knees who underwent the MRI using a different protocol were excluded. The study was approved by our institutional review board.

The size of the femoral component was determined intraoperatively, using the medial femoral condyle sizer (spoon) to restore the ligament tension. The senior surgeon relied on the spoon for sizing the femoral component by determining the discrepancy among the three.

**Figure 1:**
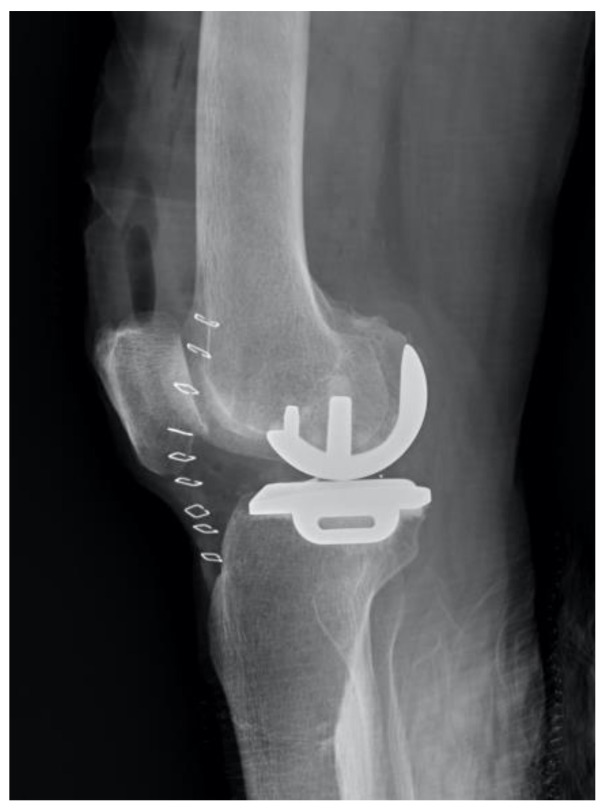
A postoperative lateral view of the femoral component underhang.

**Figure 2:**
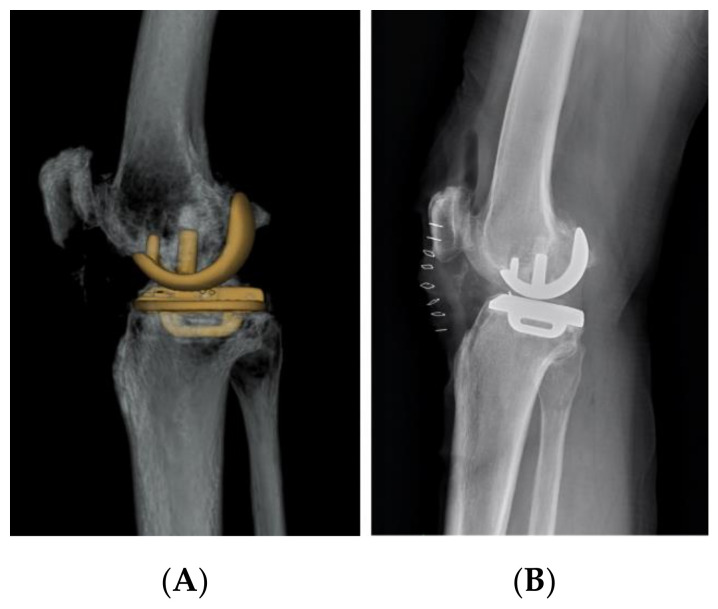
An example of ideal femoral component size of the knee lateral view and the same patient in CT scan showed a little overhang from posterior condyle bony contour. (**A**) 3D image. (**B**) Standard lateral view.

**Figure 3:**
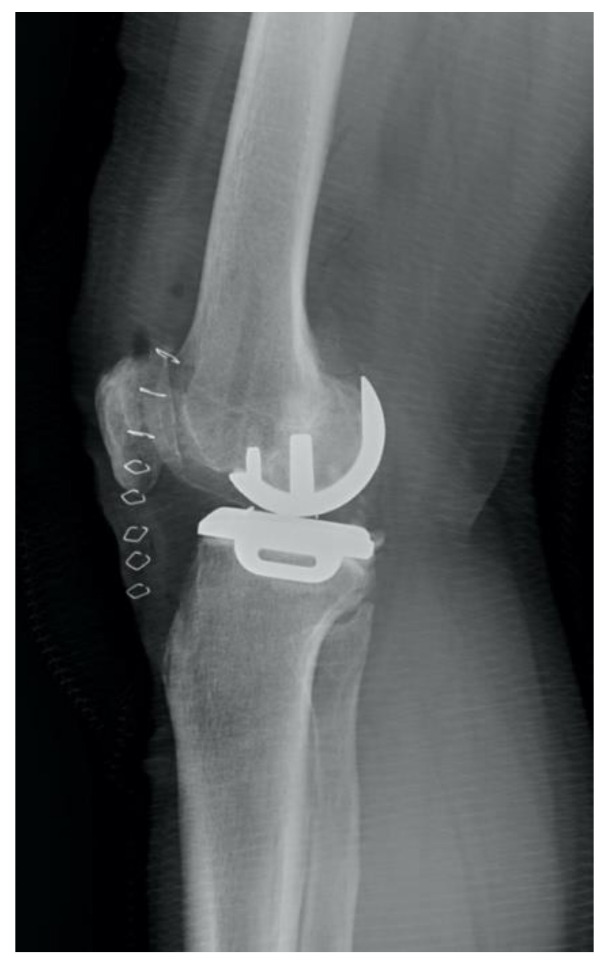
A postoperative image of the femoral component overhang over 4 mm.

The component sizing was assessed on postoperative true lateral radiographs according to the criteria laid down by the Oxford Group. We arranged the postoperative radiograph immediately after the operation, while the patients were still anesthetized. As the result we could get the ideal lateral radiographs. Good postoperative radiographic assessment was referenced from the standard Oxford operation manual as a baseline for comparison. Components that were flush with the posterior condyle or smaller were considered underhang (Figure 1), up to a 2 mm overhang beyond the bony contour of the posterior margin of the medial femoral condyle was considered ideal (Figure 2), and posterior overhang of >2 mm was considered inappropriate (Figure 3).

All MRI examinations were performed using a 3-Tesla MRI scanner (Discovery MR750; GE Medical Systems, Milwaukee, WI, USA) with a knee coil (GE 3T HD T/R Knee Array). Patients were scanned in the supine position with the knee fully extended. The institutional MRI protocol for evaluating knee osteoarthritis consisted of proton density-weighted fast relaxation fast spin echo with fat suppression in the axial, coronal and sagittal planes; T1-weighted fast spin echo without fat suppression in the coronal plane; and 3D T2*-weighted sequence in the sagittal plane. Sagittal images were obtained in a plane perpendicular to a line connecting the posterior femoral condyles on axial images and the tibial plateau on coronal images. The parameters for proton density-weighted images were as follows: repetition time/echo time, 2500/30 ms; rectangular field of view, 160 × 160 mm^2^; slice thickness, 4.0 mm; space, 1.0 mm; matrix, 352 × 256; NEX, 3; echo train length, 7; bandwidth, 35.71. All measurements were performed on a picture archiving and communication system (Centricity Enterprize Web V3.0; GE Healthcare, Barrington, IL, USA).

**Figure 4:**
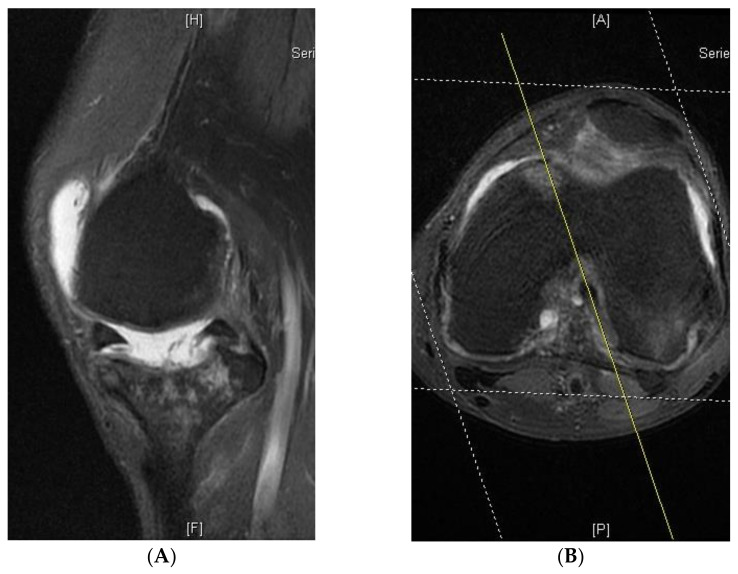
An unideal sagittal plane axis of magnetic resonance imaging would lead to incorrect measurement. (**A**) Sagittal cut of the MRI image. (**B**) Axial cut of the MRI image. The yellow line is the axis of the sagittal cut.

Retrospectively, MRI was used to measure the true AP (anterior-posterior) diameter of the medial femoral condyle. Examinations were excluded if they were of poor quality or if anatomical abnormalities prevented adequate characterisation of the anatomical structures used for measurements, including the deepest point of the trochlea, posterior condyles of the femur and posterior cruciate ligament (PCL). In addition, if the sagittal plane was not aligned with the abovementioned axis, these images could not be used for femoral component measurement (Figure 4A,B).

**Figure 5:**
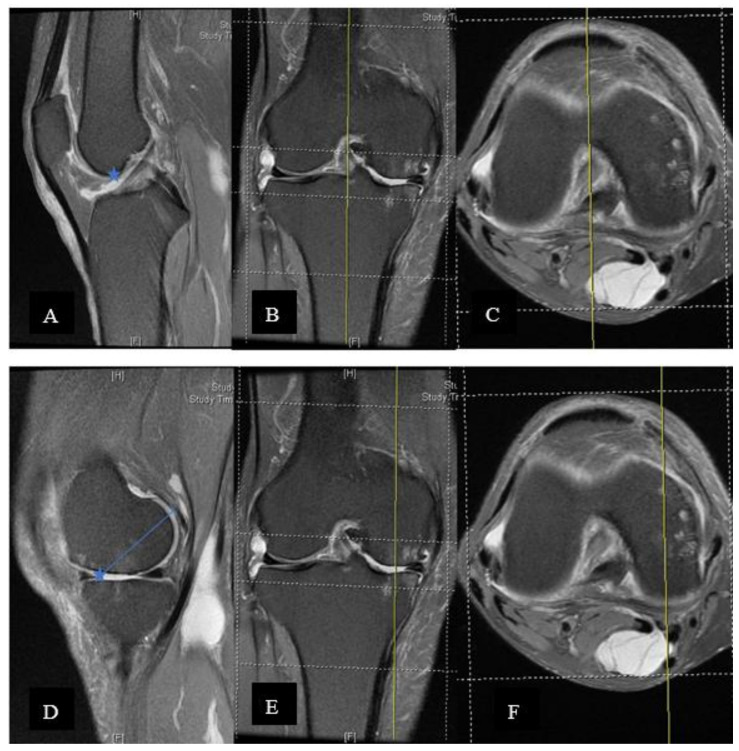
(**A**–**C**) On the sagittal section, the cut that bisected the trochlea was obtained, confirming the axial and coronal sections. The corner between trochlea and blummensant line was identified and marked (star). (**A**) Central sagittal cut of the knee MRI. (**B**) Coronal cut of the knee MRI. (**C**)Axial cut of the knee MRI. (**D**–**F**) The marked point was transferred to the sagittal section at the central cut of the medial femoral condyle (also have to check on the axial and coronal planes). The marked point would estimate the most anterior point of the medial femoral condyle. The most posterior point included the visualised and marked cartilage. A full thickness loss of cartilage is usually observed at the anterior region. The cartilage thickness should be added back. (**D**) Sagittal cut through medial femoral condyle of the knee MRI (**E**) Coronal cut of the knee MRI (**F**) Axial cut of the knee MRI.

The medial femoral condyle AP diameter measurements were obtained as described below: On the sagittal section, the cut that bisected the trochlea was obtained and confirmed on axial and coronal sections. The corner between the trochlea and blummensant line was identified and marked. The marked point was then transferred to the sagittal section at the central cut of the medial femoral condyle and was used to estimate the most anterior point of the medial femoral condyle. The most posterior point including cartilage is then marked. Usually, a full thickness loss of the cartilage is observed in the anterior region. The cartilage thickness should be added back (we used the lateral cartilage thickness as the estimation) (Figure 5 A–F).

Three different height- and gender-based criteria [6,11,12], as well as X-ray templating according to the Oxford^®^ Partial Knee Manual were used, and the accuracy of each criterion was calculated. All the measurements were done and rechecked by three physicians (C.-P. Yang, Y.-C. Lai, K.-Y. Hsu).

### Statistical Analysis

We used the Statistical Package for the Social Sciences (IBM SPSS Statistics 23.0, IBM, Armonk, NY, USA) and Microsoft Office Excel (Microsoft Office 2016). All categorical data were analyzed using Fisher’s exact test. Results were considered statistically significant at a *p*-value < 0.05.

## 3. Results

There were 17 men (18 knees) and 33 women (34 knees). The overall mean height of the cohort was 157.1 cm (standard deviation (SD) 7.7; range 144–172 cm), whereas the mean height of males and females was 165.3 cm (SD 7.6 cm; range 158–178 cm) and 152.8 cm (SD 7.7; range 144–162 cm), respectively.

The results of each measurement method are listed in Table 1. Intraoperatively, surgeons mainly used the spoon for femoral implant sizing. The size was checked on postoperative radiographs to determine the accuracy of the spoon, and the accuracy was 67.3%. Overhung was found in 2 knees (3.8%) and underhung in 15 knees (28.8%). Two cases were of 2 sizes out. The ideal size for each knee was recorded.

According to the Oxford algorithm, the prediction of the femoral implant size was correct in 42.3% of patients. A total of 29 cases were underhang and 1 case was overhang. Two cases were 2 sizes out. The Chinese algorithm by Tu et al. in the Chinese population and Indian algorithm by Malhotra et al. have an accuracy of 23% and 25%, respectively. Following Tu’s algorithm, 35 cases were underhung and 5 cases overhung. Among these cases, up to 10 cases were of 2 sizes out. Using Malhotra’s criteria for the Indian population, all incorrect cases were underhung and 11 cases were 2 sizes out.

Conversely, X-ray templating has an accuracy of 42.3%: 10 of the unideal cases were underhung, and 20 cases were overhung. Among them, 3 cases were of 2 sizes out.

In contrast, using MRI to estimate the femoral implant size has an accuracy of 90.3%. Five incorrect cases were all overhang and one size out. This prediction method was significantly more accurate than all the above-mentioned methods (Table 2).

## 4. Discussion

UKA is becoming more widely used because of its approximate knee kinematics. According to previous studies, UKA walked 10% faster than TKA. In addition, stride length and stance time are both much closer to normal than TKA [19,20]. However, no reliable and universal method was available to correctly select the femoral component in all kinds of patients. In this study, we compared all existing criteria in selecting femoral component size, such as radiographic templating and spoon. We also included three height- and gender-based criteria established by Fawzy et al., Tu et al. and Malhotra et al.

The precision and accuracy of the templating system had already been studied by Kasis et al. in 2004. They suggested a high level of intra-observer reproducibility for preoperative templating. The inter-observer agreement was poor, which may be due to a lack of reliable anatomical landmarks on two-dimensional radiographs [13]. Bothra et al. also questioned the reliability of preoperative radiological templating in UKA [7]. Conversely, Fawzy et al. examined the accuracy of templating and found that the femoral component prediction was correct in 67% of cases. All incorrect cases were one size different from the ideal [6]. In our study, the templating system was correct in 42.3% of patients; however, 3 of them were 2 sizes out. The main shortcoming may be due to variable magnification of the bony contour in the radiographic image. The ideal magnification should be 105% if a template is used. Second, distinguishing the medial and lateral condyles may be difficult if the image quality was poor (e.g., the limb may be mal-rotated).

The femoral sizing spoon was designed for the facilitation of microplasty instrumentation. The proper size of the spoon should fit the medial condyle and restore the proper ligament tension. In some situations, posterior osteophytes or partial-thickness cartilage loss are present, which may lead to an incorrect size. Malhotra et al. used the sizing spoon to decide the femoral component intraoperatively. Their study showed that the overall accuracy was 74.6% [12]. For our patients, we relied on the sizing spoon at the index surgeries. Retrospectively, we found that the accuracy was 67.3%. Therefore, we proposed an MRI-based measurement that can accurately and universally obtain the ideal size pre-operatively.

According to Fawzy et al., height and gender can accurately predict the ideal size in 75% of patients, whereas it was one size out in 25% of patients and no patient had more than one size out [6]. Using the same criteria, the ideal size of the femoral component could only be achieved in only 42.3% of our patients. Malhotra et al. obtained an ideal size of 74% using their modified guideline, but only 25% in our study.

In a study published by Tu et al., a C-arm intensifier guide (CAIG) was used to determine the size of each femoral implant intra-operatively, which had an accuracy of 92%. They also set a modified algorithm for the Chinese population. Using the Chinese algorithm, they have greater accuracy of prediction (88%) than the Oxford algorithm (51.1%) [11]. Although our patients were quite the same ethnic groups, this algorithm only achieved 23% correct femoral size in our patient group. We only deduced in the past that the knee anatomy differs between Caucasian and Asian population groups [21,22,23]. However, even in similar populations, height- and gender-based criteria seemed to have unsatisfactory accuracy. Indeed, the C-arm intensifier guide (CAIG) may have precisely estimate the femoral component. However, the radiation exposure would increase and the infection rate with this kind of setting is unknown. Furthermore, the femoral component trail should be installed first using Tu’s technique. In this step, both tibial and femoral bone cuts were performed. Hence, it is not practical in clinical situations. In addition, we did not use tibial implant size as a predictor for femoral component size because of the same reasons mentioned by Malhotra et al. [12]. First, femur sizing is performed before tibial size determination intra-operatively, and femur sizing would affect the tibial cutting level. Second, the tibial component size can change intra-operatively if tibial re-cut is required.

The importance of correct femoral implant size is still debated [24]. As we know, Phase I and Phase II of the Oxford medial UKA still had a 20-year survival rate of 92%, which is similar to the best reported TKR survival [6]. Even though these two generations had only one size femoral component, which is similar with the medium size of the current inventory. The clinical outcome in these cases was also not compromised. Therefore, it is safe to assume that an acceptable femoral size is one that is ideal or one size out. There is no data to compare the clinical outcomes of patients with ideal and incorrect femoral component size; therefore, further study with long-term follow-up is necessary.

The role of MRI in Oxford UKA has not yet been established [14,15]. Common radiographic examination for its suitability includes anteroposterior, true lateral, varus and valgus stress and patellar axial views. Hamilton et al. designed a decision aid algorithm composed of the abovementioned imaging study and presented improved Knee Society Score and implant survival for patients who met the criteria of the decision aid, compared to those who did not meet the criteria [25]. Hamilton et al. also argued that full-thickness cartilage loss or subchondral edema showed in MRI did not significantly affect the functional outcome in patient with partial-thickness cartilage loss [14]. Tuecking et al. also concluded that the appropriateness of UKA could be achieved with the same radiographic decision aid. In their results, the suitable UKA showed a higher clinical outcome score (Knee injury and osteoarthritis outcome score-quality of living (KOOS-QDL): 68.75) and lower revision rate (7.3%) compared to unsuitable UKA (50.0%; KOOS-QDL: 50.0). Nevertheless, their data showed an overall sensitivity (70.1%) and specificity (76.2%) for the criteria. Approximately 22.7% false negatives were found to result exclusion UKA implantation [17]. Jacobs et. al. once reported the bone marrow lesions in the tibia and the medial compartment related to better postoperative functional improvement [26]. Our institution conducted an MRI study before the Oxford UKA to evaluate the true cartilage loss, meniscal root lesion and ACL condition. Retrospectively, we designed a measurement protocol based on MRI that could accurately select an ideal size for the femoral component. In our study, the accuracy using this protocol reached 90.3%, whereas no cases are 2 sizes out. As a result, we proposed a new application for MRI studies before Oxford UKA. This is the first study using MRI to measure the medial femoral condyle diameter and determine femoral component size.

The main limitation of this study is the small number of patient groups. As a result, the reliability of this method is questionable. In addition, we excluded some patients due to oblique cut of sagittal views (Figure 4), which may result in incorrect measurements. The exact range of sagittal view obliquity requires a more precise investigation.

## 5. Conclusions

The existing algorithms for selecting femoral component size in Oxford UKA yields variable results in different studies and ethnic groups. We proposed an accurate method based on knee MRI that can be applied universally. This may strengthen the importance of MRI in UKA planning. However, the true validity of this method remains to be confirmed in a larger patient group.

## Figures and Tables

**Table 1:** Accuracy of template, sizing spoon, previous guidelines and MRI measurement.

	Correct	Incorrect	Accuracy of Prediction
Templating	22	30 (3 two size out)	42.3%
Spoon	35	17 (2 two size out)	67.3%
Fawzy et al. [6]	22	30 (2 two size out)	42.3%
Tu et al. [11]	12	40 (10 two size out)	23%
Malhotra et al. [12]	13	39 (11 two size out)	25%
MRI measurement	47	5 (0 two size out)	90.3%

**Table 2:** Statistical analysis comparing different methods.

	*p*-Value (Fisher’s Exact Test)
MRI versus templating	<0.001
MRI versus spoon	0.007
MRI versus Fawzy et al.	<0.001
MRI versus Tu et al.	<0.001
MRI versus Malhotra et al.	<0.001

## Data Availability

The dataset supporting the conclusions of this article is available from the corresponding author on reasonable request.
